# General practitioner prescribing of single and combination nicotine replacement therapy in the UK: a retrospective database study

**DOI:** 10.1186/1471-2296-15-47

**Published:** 2014-03-20

**Authors:** Michelle Johnson, Pippa Anderson, Ian Lockhart

**Affiliations:** 1 Cegedim Strategic Data Medical Research Ltd, London, UK; 2 Swansea Centre for Health Economics, Swansea University, Wales, UK; 3 Pfizer Ltd., Tadworth, Surrey, UK

**Keywords:** Nicotine replacement therapy, Smoking cessation, The health improvement network, Observational research

## Abstract

**Background:**

Guidance in England and Wales recommends that nicotine replacement therapies (NRTs), varenicline or bupropion should be offered for smoking cessation support. Research on general practitioner (GP) NRT prescribing patterns for smoking cessation is lacking in the published literature.

**Methods:**

UK primary care electronic health records were retrospectively analysed to identify the most common GP initiated NRT prescribing patterns, characterise people who receive NRT and determine whether NRTs given in a first quit attempt are different from subsequent attempts.

**Results:**

The study population comprised 38,954 individuals in UK primary care data with a first ever NRT patch smoking cessation attempt for the period January 2008-December 2011. The majority (64.3%) received NRT patch monotherapy at first smoking cessation attempt, and the most common NRT was 21 mg/24 hours patch monotherapy (15.2%). Of the 35.7% first smoking cessation attempts which were NRT combination therapy, the most common combination was patch + inhalator (56.2%). The proportion of people who started a smoking cessation attempt with combination therapy increased from 25.7% in 2008 to 44.8% in 2011. The majority of the population had one recorded smoking cessation attempt but a significant minority (20.2% N = 7,868) started a second smoking cessation attempt. Second and third attempts, while predominantly patch monotherapy, also demonstrated an increasing use of NRT combinations over the study period (2^nd^episode: 20.6%-38.2%; 3^rd^episode: 20.0%-36.8%). However, a minority received only non-patch NRT during second and third NRT episodes. Taking into account the 39,068 people prescribed NRT patch during the study period with a history of NRT at baseline (excluded from the analysis), the total proportion of people prescribed NRT patch between 2008–2011 who had more than one NRT episode was 48.4% (46,936/96,986) and of 128,115 NRT users, only 14.7% (N = 18,838) were prescribed bupropion or varenicline prior to NRT use.

**Conclusions:**

The study findings represent new data describing GP NRT prescription patterns in the UK. Given the predominance of NRT patch monotherapy observed, health policy makers and service commissioners should ensure that GPs provide equality of access to all recommended smoking cessation pharmacotherapies.

## Background

Smoking is a major cause of preventable illness and premature death – estimates for 2009 suggest that in England smoking led to 81,400 premature deaths and treating smoking-related illness cost the UK National Health Service (NHS) 2.7 billion in 2006 [1].

The tobacco control plan for England [2] supports the NHS in providing smoking cessation services and encourages commissioners to put local schemes in place and improve services, while the UK Quality and Outcomes Framework (QOF) [3] provides incentives for GPs to enable people to access smoking cessation support.

Most smoking cessation services in the UK are provided in primary care and delivered by a variety of health care professionals in differing settings. These include specialist smoking cessation services, community pharmacies, and GP surgeries. The National Institute for Health and Care Excellence (NICE) guidance supports health professionals as they undertake smoking cessation interventions in their practices. Public health guidance issued by NICE [4] recommends that NRTs, varenicline or bupropion should be offered to people who are planning to stop smoking.

NICE Public Health Guidance 10 Recommendation 4 (4) directs health professionals as follows:

•Offer NRT, varenicline or bupropion, as appropriate, to people who are planning to stop smoking.

•Offer advice, encouragement and support, including referral to the NHS Stop Smoking Service, to help people in their attempt to quit. NRT, varenicline or bupropion should normally be prescribed as part of an abstinent-contingent treatment, in which the smoker makes a commitment to stop smoking on or before a particular date (target stop date). The prescription of NRT, varenicline or bupropion should be sufficient to last only until 2 weeks after the target stop date. Normally, this will be after 2 weeks of NRT therapy, and 3–4 weeks for varenicline and bupropion, to allow for the different methods of administration and mode of action. Subsequent prescriptions should be given only to people who have demonstrated, on re-assessment, that their quit attempt is continuing.

•Explain the risks and benefits of using NRT to young people aged from 12 to 17, pregnant or breastfeeding women, and people who have unstable cardiovascular disorders. To maximise the benefits of NRT, people in these groups should also be strongly encouraged to use behavioural support in their quit attempt.

•Neither varenicline or bupropion should be offered to young people under 18 nor to pregnant or breastfeeding women.

•Varenicline or bupropion may be offered to people with unstable cardiovascular disorders, subject to clinical judgement.

•If a smoker's attempt to quit is unsuccessful using NRT, varenicline or bupropion, do not offer a repeat prescription within 6 months unless special circumstances have hampered the person's initial attempt to stop smoking, when it may be reasonable to try again sooner.

•Do not offer NRT, varenicline or bupropion in any combination.

•Consider offering a combination of nicotine patches and another form of NRT (such as gum, inhalator, lozenge or nasal spray) to people who show a high level of dependence on nicotine or who have found single forms of NRT inadequate in the past.

•Do not favour one medication over another. The clinician and patient should choose the one that seems most likely to succeed.

•When deciding which therapies to use and in which order, discuss the options with the client and take into account:

○ whether a first offer of referral to the NHS Stop Smoking Service has been made

○ contra-indications and the potential for adverse effects

○ the client's personal preferences

○ the availability of appropriate counselling or support

○ the likelihood that the client will follow the course of treatment

○ their previous experience of smoking cessation aids.

NRTs are available in a number of differing formulations and delivery mechanisms to enable tailored smoking cessation attempts. NICE guidance [4] suggests that prescribers match products to client lifestyles and preferences. Furthermore, the guidance recommends that combinations of nicotine patches and another form of NRT (such as gum, inhalator, lozenge or nasal spray) should be offered to people who show a high level of dependence on nicotine or who have found single forms of NRT inadequate in the past [4].

If NRT is the smoking cessation strategy chosen, the nicotine patch which releases a small but constant stream of nicotine into the blood-stream is the recommended ‘anchor’ treatment to which other, faster-acting, NRTs are added [4]. Previous research using data from the UK specialist smoking cessation service has evaluated intervention characteristics and success rates, and published data on first-line monotherapy NRT and combination NRT use [5]. However, there is no published research on GP prescribing of NRT, and to date there is no UK research that describes which NRT combinations are most frequently used and when, both at initial and in subsequent quit attempts.

In the UK routinely collected patient record data are available for analysis to aid understanding of ‘real life’ use. Access to this data enabled a retrospective study of NRT GP prescribing patterns to be conducted. The aim of the study was to describe the prevalence of NRT patch use and the types of NRT combinations involving a patch that are routinely prescribed in clinical practice by GPs. This was conducted by scrutinising the routinely collected primary care data from GP surgeries between 1st January 2008 (NICE public health guidance on smoking cessation services [4] was published in February 2008) and 31st December 2011.

## Methods

### Research objectives

#### The specific objectives of this study were to

1. Identify the most commonly used NRT patterns of treatment involving a patch and rates of use among GPs in UK primary care;

2. Characterise people who receive NRT patch and NRT patch combinations;

3. Determine whether the NRTs given as the first NRT smoking cessation attempt is different from those used for subsequent attempts.

### Study design

Pseudo-anonymised patient data are collected in a non-interventional setting from the daily records of general practices which use the Vision practice management software in the UK and have agreed to contribute data to The Health Improvement Network (THIN). As of January 2013, the THIN database contained primary care medical records from over 11.7 million patients, of which over 3.8 million are actively registered, from 570 general practices. The age and gender profile of the active patient population in THIN has been shown to be comparable to the UK population [6-8]. Analysis is regularly undertaken to compare general population distributions and demographics with those of THIN and incidence of key diseases has also been compared to UK clinical practice with very good correlations. The THIN database has been validated for monitoring prescriptions for smoking cessation medications [9].

### Data collection

This is a retrospective observational study using routinely collected primary care electronic health records from UK general practices. The study subjects were required to have at least 6 months of quality-controlled baseline data (between index date and the later of patient registration date, practice Vision date [date the practice started using the computer software Vision] and Acceptable Mortality Reporting [AMR] date). The AMR date is a data quality parameter defining the period for which mortality reporting in the practice is deemed complete [10]. Baseline information for each patient was based on data from the start of patient records through to the first ever NRT patch prescription (index date).

**Figure 1:**
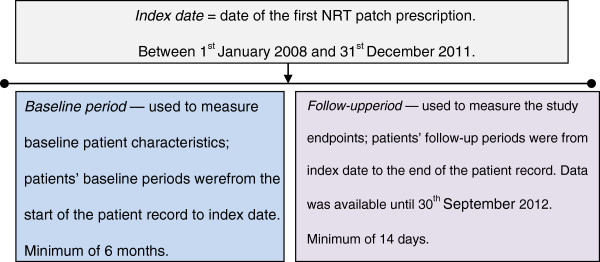
Study design and duration.

Follow-up information for each patient was based on data from the index date (inclusive) to the end of patient records (earliest date of patient transfer out of practice and practice last data collection date). Study subjects were required to have at least 14 days of follow-up data. Figure 1 illustrates the study design.

### Study population

The study population consisted of individuals identified within the THIN database with a first ever (incident) smoking cessation attempt with an NRT patch prescription reported between the study period dates of 1st January 2008 and 31st December 2011. The inclusion and exclusion criteria for the study are described below.

#### Inclusion criterion

•NRT patch prescription during the study period (1st January 2008 and 31st December 2011).

#### Exclusion criteria

•Under 18 years old at first NRT patch prescription during the study period (to be in line with NRT prescribing for the adult population eligible for NRT, varenicline or bupropion as advised in the NICE PH10 guidance).

•Less than 6 months of quality-controlled data prior to first NRT patch prescription during the study period (would not allow sufficient data to assess baseline characteristics).

•Less than 14 days in the database after first NRT patch prescription during the study period (would not allow sufficient follow-up data to assess the course of NRT treatment).

•History of NRT patch products prior to first NRT patch prescription during the study period.

•History of a non-patch NRT product up to two weeks prior to first NRT patch prescription during the study period (ensures that the study focuses on and follows-up people who received NRT prescribed for first-line smoking cessation therapy, including those who receive non-patch NRT in the two weeks prior to an NRT patch to allow for the possibility of combination therapy).

•History of bupropion or varenicline prior to first NRT patch prescription during the study period.

#### Characteristics of patient population

Baseline characteristics were collected for all study subjects prior to index date and information on the prescription of NRT was collected during follow-up.

#### Defining NRT episodes

The first NRT episode was defined as starting on the date of the index date. From this starting point, going forward in chronological order, all the NRT prescriptions were grouped into episodes using the following rule: the gap between the previous prescription and the next prescription had to be less than or equal to 90 days to qualify as the same episode. If the gap between prescriptions was greater than 90 days, this was defined as a new NRT episode. All first NRT episodes had to be between 2008 and 2011, however some subsequent (2nd and 3rd) episodes started in 2012 and hence are reported in the results.

The initial NRT included all products and strengths prescribed during the first NRT episode. An interval of more than 90 days between the end of the last NRT episode during follow-up and end of follow-up, i.e. a patient who received no further NRT prescriptions during the last 90 days, indicated that the patient was no longer receiving prescribed NRT as smoking cessation support. Study subjects who were considered *not* to have discontinued all NRT were those whose last NRT episode ended during the 90 days before the end of follow-up, or on or after the end of follow-up. The reason for discontinuation – whether the smoking cessation attempt was successful or not – is not available in the data.

### Data analysis

The analysis comprised descriptive statistics; categorical data were summarised by the number and percentage of people in each category, and continuous data were summarised by the number of people and minimum and maximum values. Mean, standard deviation, ranges, median and lower and upper quartiles were reported. Baseline characteristics for all study subjects and according to initial NRT were summarised. Further details summarised included the frequency of use and details of each NRT within each episode of treatment, duration of baseline and follow-up time, and the average number and percentage of people who started a subsequent episode or discontinued therapy during follow-up according to initial NRT episode. All data management was performed using SAS (version 9.2).

### Ethical conduct of the study

The study was conducted in accordance with legal and regulatory requirements, as well as with scientific purpose, value and rigor and follows generally accepted research practices described in Good Pharmacoepidemiology Practices issued by the International Society for Pharmacoepidemiology and Good Epidemiological Practice guidelines issued by the International Epidemiological Association. The data collection scheme for THIN is approved by the UK Multicentre Research Ethics Committee (reference number: 07H1102103). In accordance with this approval, the study protocol was reviewed and approved by an independent Scientific Review Committee (SRC) (reference number: 13–011).

## Results

Over 128,000 people were initially identified with a prescription for an NRT product (patch or non-patch) during the study period. Approximately a quarter (31,129 people - 24.3%) received a non-patch NRT product alone as their smoking cessation treatment in this period. Of these, 11,320 people (36.4%) had a history of NRT products (patch or non-patch) prior to the study period and 5,049 people (16.2%) had a prior history of bupropion or varenicline as their smoking cessation treatment. The majority of the non-patch NRT study subjects were prescribed either an inhalator only (50.6%), gum only (16.8%) or a combination of more than one type of non-patch NRT (13.6%). Since this cohort received only a non-patch NRT, they were excluded. This left 96,986 people with an NRT patch product during the study period.

**Figure 2:**
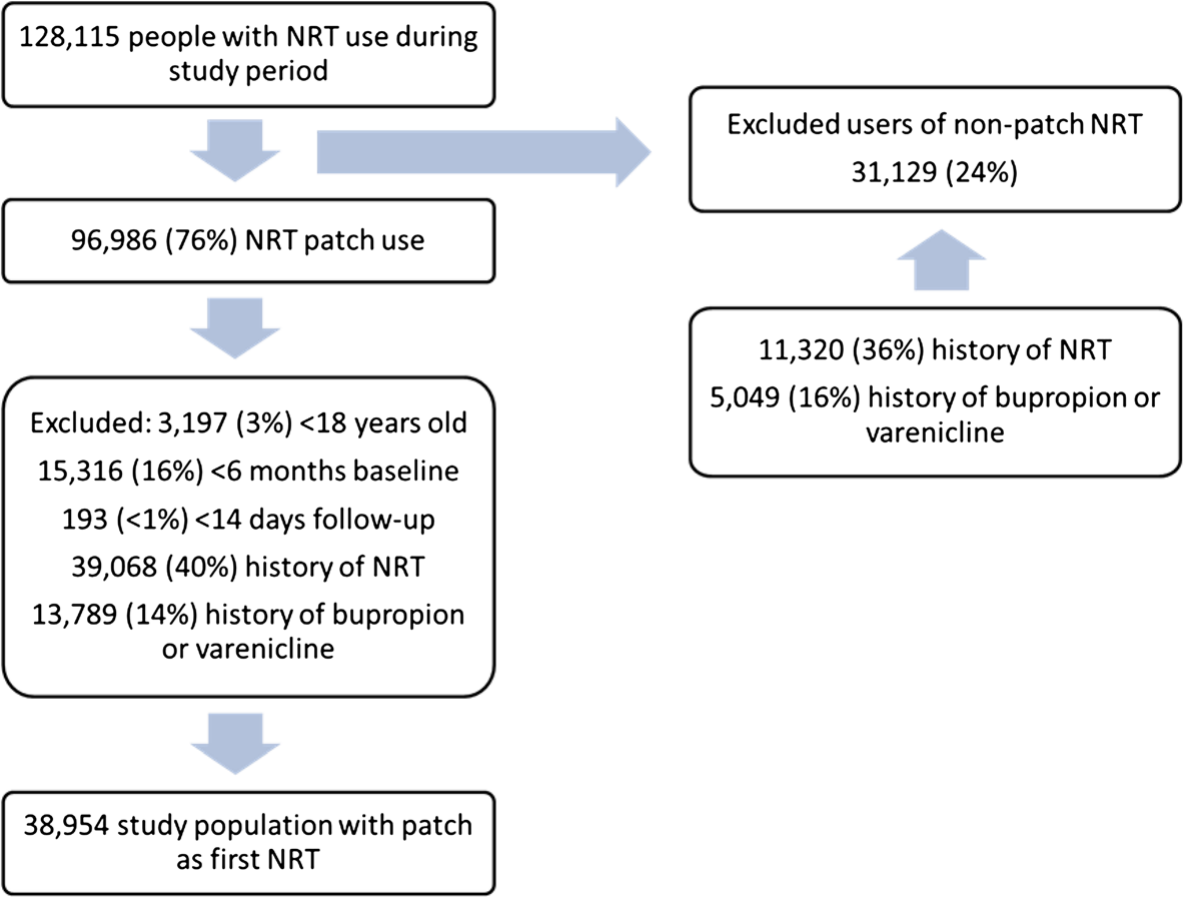
Overall patient selection process.

Of the 96,986 people who had NRT patch as their smoking cessation treatment, 38,954 adults were identified with a first ever NRT patch prescription in the study period between 1st January 2008 and 31st December 2011. The criterion which excluded the largest proportion of people (40.3%) was a history of NRT products. A further 14.2% received bupropion or varenicline prior to their first NRT patch in the study period and were also excluded. Figure 2 illustrates the selection process and Table 1 gives more details. Further descriptive statistics on NRTs prescribed are also provided in Table 2.

**Table 1:** Study population selection process

**Selection process**	**n**	**%**
*Inclusion:* people with an NRT product during the study period	128,115	100.00
*Exclusion:* people with NRT non-patch products only during the study period	31,129	24.30
*NRT non-patch products (N = 31,129):*		
NRT gum only	5,228	16.79
NRT tablet only	1,187	3.81
NRT inhalator only	15,750	50.60
NRT lozenge only	3,631	11.66
NRT oral spray only	589	1.89
NRT nasal spray only	527	1.69
More than one type of NRT non-patch	4,217	13.55
People with NRT patch product during the study period	96,986	75.70
*Exclusions (N = 96,986)*^a^		
Under 18 years old	3,197	3.30
Less than six months of baseline data	15,316	15.79
Less than fourteen days of follow-up data	193	0.20
History of NRT products (patch or non-patch)^b^	39,068	40.28
History of bupropion or varenicline	13,789	14.22
**Study population**	38,954	30.41

^a^Based on date of first NRT patch during the study period. Patients were not excluded in a cascade manner, therefore some patients met more than one of the exclusion criteria. A total of 58,032 patients with an NRT patch product were excluded.

^b^History of NRT patch product any time prior to index date (date of first patch during study period) or history of NRT non-patch product any time before two weeks prior to index date.

**Table 2:** Initial NRT (all products during first NRT episode) of study population, according to year of identification

**Initial NRT**	**Study period (2008–2011)**		**2008**		**2009**		**2010**		**2011**	
	**N=**	**38,954**	**N=**	**10,632**	**N=**	**10,501**	**N=**	**9,337**	**N=**	**8,484**
	**n**	**%**	**n**	**%**	**n**	**%**	**n**	**%**	**n**	**%**
Patch only	25,046	64.30	7,895	74.26	6,940	66.09	5,530	59.23	4,681	55.17
Patch and gum	2,136	5.48	528	4.97	617	5.88	526	5.63	465	5.48
Patch and tablet	411	1.06	108	1.02	107	1.02	124	1.33	72	0.85
Patch and inhalator	7,822	20.08	1,522	14.32	2,103	20.03	2,252	24.12	1,945	22.93
Patch and lozenge	1,593	4.09	298	2.80	360	3.43	451	4.83	484	5.70
Patch and oral spray	270	0.69	0	0.00	0	0.00	0	0.00	270	3.18
Patch and nasal spray	129	0.33	35	0.33	25	0.24	33	0.35	36	0.42
≥ 3 types of NRT	1,547	3.97	246	2.31	349	3.32	421	4.51	531	6.26

The follow-up time for the people included in the study ranged between 15 days and 4.7 years with median 2.5 years (lower and upper quartiles 1.5 to 3.6). On average, in our findings, there was one year between NRT episodes (for those people who had more than one NRT episode) with a range of 91 to 1693 days.

Table 3 provides descriptive statistics for the study population. The study population was equally distributed between males (49.7%) and females. The three most prevalent co-morbidities in the study population – asthma (14.9%), chronic obstructive airways disease (COPD) (7.6%) and cardiovascular disease (CVD) (6.5%) – had higher rates than the general population [2]. For example, the national prevalence of asthma, COPD and CVD in 2011/12 in England was 5.9%, 1.7% and 1.7% respectively [2]. The differences are most likely an artefact of our data owing to the comparison with the general population which includes non-smokers and potentially a greater incentive for people with these co-morbidities to stop smoking.

**Table 3:** Baseline characteristics of study population according to initial NRT taken

**Characteristic**	**All people**		**Patch only**		**Patch and gum**		**Patch and tablet**		**Patch and inhalator**		**Patch and lozenge**		**Patch and oral spray**		**Patch and nasal spray**		**≥3 types NRT**	
	**N=**	**38,954**	**N=**	**25,046**	**N=**	**7,822**	**N=**	**411**	**N=**	**7,822**	**N=**	**1,593**	**N=**	**270**	**N=**	**129**	**N=**	**1,547**
Age (years)																		
Minimum, maximum	18.00	96.00	18.00	96.00	18.00	86.00	18.00	83.00	18.00	91.00	18.00	88.00	18.00	80.00	18.00	77.00	18.00	84.00
Mean, standard deviation	42.95	15.74	42.89	16.04	40.79	14.31	44.27	15.06	43.13	15.28	44.10	15.56	40.03	14.47	43.04	14.19	44.92	15.21
Median	42.00		41.00		40.00		44.00		42.00		44.00		39.00		42.00		44.00	
Lower quartile, upper quartile	30.00	55.00	29.00	55.00	29.00	51.00	32.00	55.00	30.00	55.00	31.00	56.00	27.00	52.00	32.00	54.00	32.00	58.00
Gender (n,%)																		
Male	19,373	49.73%	12,622	50.40%	1,140	53.37%	193	46.96%	3,688	47.15%	796	49.97%	135	50.00%	81	62.79%	718	46.41%
Female	19,581	50.27%	12,424	49.60%	996	46.63%	218	53.04%	4,134	52.85%	797	50.03%	135	50.00%	48	37.21%	829	53.59%
Co-morbidities (n,%)																		
Cardiovascular disease	2,538	6.52%	1,687	6.74%	94	4.40%	27	6.57%	490	6.26%	82	5.15%	14	5.19%	7	5.43%	137	8.86%
Asthma	5,787	14.86%	3,647	14.56%	318	14.89%	58	14.11%	1,211	15.48%	216	13.56%	49	18.15%	23	17.83%	265	17.13%
Chronic obstructive airways disease	2,944	7.56%	1,838	7.34%	121	5.66%	34	8.27%	631	8.07%	135	8.47%	22	8.15%	8	6.20%	155	10.02%
Diabetes (type I or II)	1,816	4.66%	1,096	4.38%	107	5.01%	18	4.38%	393	5.02%	89	5.59%	11	4.07%	7	5.43%	95	6.14%
Stroke	540	1.39%	371	1.48%	22	1.03%	6	1.46%	96	1.23%	14	0.88%	1	0.37%	1	0.78%	29	1.87%

The Townsend score of social deprivation indicates that the study population tended to live in less affluent areas. Overall, 46.6% (25.0% and 21.6%) of study subjects lived in the least affluent areas (Townsend quintile scores 4 and 5 respectively) while only 31.6% (14.8% and 16.8%) lived in the most affluent areas (Townsend quintile scores 1 and 2 respectively). This differs from the distribution of the overall THIN population as of May 2012, in which 32.0% lived in the least affluent areas (18.9% and 13.1%, Townsend quintile scores 4 and 5 respectively) and 46.9% lived in the most affluent areas (25.2% and 21.7%, Townsend quintile scores 1 and 2 respectively).

Current guidelines recommend that pregnant or breastfeeding women are prescribed NRT if they are unable to stop smoking without pharmacotherapy [11]. In this study we observed that of the 1,786 women (9.1%) who were pregnant, the majority (63.4%) received NRT patch only as part of their first smoking cessation episode. The next most prevalent initial NRT for pregnant women was patch and inhalator (21.7%) and patch and gum (5.4%). Very few pregnant women (10 women, 0.6% of pregnant women) were prescribed an NRT in tablet form in their initial NRT episode.

The majority (64.3%) of people received a NRT patch as monotherapy at their first attempt at smoking cessation. The most frequent NRT patch prescription regimens are presented in Table 4. The most common regimen was 21 mg/24 hours patch only (15.2% of all people). Other commonly prescribed monotherapy patch regimens included 15 mg/16 hours patch only (9.7%), 14 mg/24 hours patch only (5.6%) and 10 mg/16 hours patch only (3.8%).

**Table 4:** The ten most frequent treatment pathways of study population (N = 38,954)

	**First NRT episode**		**Second NRT episode**		**Third NRT episode**		**n**	**%**
	**First-line**	**Add-on**	**Second-line**	**Add-on**	**Third-line**	**Add-on**		
1	21 mg/24 hours patches	None	None	None	None	None	5,906	15.16
2	15 mg/16 hours patches	None	None	None	None	None	3,771	9.68
3	14 mg/24 hours patches	None	None	None	None	None	2,185	5.61
4	10 mg/16 hours patches	None	None	None	None	None	1,486	3.81
5	25 mg/16 hours patches	None	None	None	None	None	1,412	3.62
6	21 mg/24 hours patches & 10 mg inhalator	None	None	None	None	None	1,280	3.29
7	25 mg/16 hours patches & 10 mg inhalator	None	None	None	None	None	1,047	2.69
8	15 mg/16 hours patches & 10 mg inhalator	None	None	None	None	None	966	2.48
9	21 mg/24 hours patches	7 mg/24 hours patches & 14 mg/24 hours patches	None	None	None	None	741	1.90
10	21 mg/24 hours patches	14 mg/24 hours patches	None	None	None	None	716	1.84

Of the 36% of people prescribed NRT combination therapy at their first smoking cessation episode, the most common combinations were patch and inhalator (56.2% of people with combination therapy), patch and gum (15.4%) and patch and lozenge (11.5%). The proportion of people who started their smoking cessation attempt with combination therapy increased over time from 25.7% in 2008 to 44.8% in 2011. Table 4 shows that the most commonly used combination NRT regimens were 21 mg/24 hours patch and inhalator (3.3%), 25 mg/16 hours patch and inhalator (2.7%), and 15 mg/16 hours patch and inhalator (2.5%). These three regimens were the 6th, 7th, and 8th most common NRT regimens used in the total study population (Table 4).

The majority of the study population had only one recorded smoking cessation episode within the study period but a significant minority (20.2%; N = 7,868) did start a (prescribed) second smoking cessation episode. Second and third attempts, while predominantly patch monotherapy, follow the same pattern as first attempts with gradual increase of use of NRT combinations over time (2nd episode: 20.6% to 38.2%; 3rd episode: 20.0% to 36.8%). A minority of these people also received only non-patch forms of NRT during second and third NRT episodes. Full details can be seen in Tables 5 and 6. Furthermore, taking into account the 39,068 people prescribed NRT patch during the study period who also had a history of NRT at baseline (excluded from the current analysis), the total proportion of people prescribed NRT patch between 2008 and 2011 who had more than one NRT episode recorded in the database was 48.4% (46,936/96,986 study subjects).

**Table 5:** NRT regimens prescribed during the second NRT episode, according to year of start of episode

**NRT products**	**Total**		**2008**		**2009**		**2010**		**2011**		**2012**	
	**N=**	**7,868**	**N=**	**413**	**N=**	**1,518**	**N=**	**2,046**	**N=**	**2,421**	**N=**	**1,470**
	**n**	**%**	**n**	**%**	**n**	**%**	**n**	**%**	**n**	**%**	**n**	**%**
Patch only	3,947	50.17	287	69.49	889	58.56	1,021	49.90	1,124	46.43	626	42.59
Patch and gum	293	3.72	14	3.39	61	4.02	97	4.74	81	3.35	40	2.72
Patch and tablet	57	0.72	4	0.97	12	0.79	19	0.93	15	0.62	7	0.48
Patch and inhalator	1,159	14.73	45	10.90	206	13.57	314	15.35	373	15.41	221	15.03
Patch and lozenge	293	3.72	9	2.18	30	1.98	77	3.76	113	4.67	64	4.35
Patch and oral spray	160	2.03	0	0.00	0	0.00	0	0.00	55	2.27	105	7.14
Patch and nasal spray	22	0.28	2	0.48	3	0.20	5	0.24	7	0.29	5	0.34
Other monotherapy*	1475	18.75	41	9.93	265	17.46	405	19.79	481	19.87	283	19.25
Other dual therapy*	165	2.10	5	1.21	19	1.25	40	1.96	61	2.52	40	2.72
≥ 3 types of NRT	297	3.77	6	1.45	33	2.17	68	3.32	111	4.58	79	5.37

*Non-patch products including gum, tablet, inhalator, lozenge, oral spray or nasal spray on their own or in combination with one other non-patch product.

**Table 6:** NRT regimens prescribed during the third NRT episode, according to year of start of episode

**NRT products**	**Total**		**2008**		**2009**		**2010**		**2011**		**2012**	
	**N=**	**1,926**	**N=**	**15**	**N=**	**195**	**N=**	**449**	**N=**	**748**	**N=**	**519**
	**n**	**%**	**n**	**%**	**n**	**%**	**n**	**%**	**n**	**%**	**n**	**%**
Patch only	881	45.74	9	60.00	112	57.44	234	52.12	317	42.38	209	40.27
Patch and gum	70	3.63	0	0.00	4	2.05	12	2.67	33	4.41	21	4.05
Patch and tablet	16	0.83	0	0.00	4	2.05	2	0.45	6	0.80	4	0.77
Patch and inhalator	259	13.45	3	20.00	27	13.85	61	13.59	94	12.57	74	14.26
Patch and lozenge	83	4.31	0	0.00	4	2.05	17	3.79	38	5.08	24	4.62
Patch and oral spray	59	3.06	0	0.00	0	0.00	0	0.00	28	3.74	31	5.97
Patch and nasal spray	5	0.26	0	0.00	0	0.00	0	0.00	3	0.40	2	0.39
Other monotherapy*	434	22.53	3	20.00	37	18.97	109	24.28	166	22.19	119	22.93
Other dual therapy*	35	1.82	0	0.00	1	0.51	5	1.11	19	2.54	10	1.93
≥ 3 types of NRT	84	4.36	0	0.00	6	3.08	9	2.00	44	5.88	25	4.82

*Non-patch products including gum, tablet, inhalator, lozenge, oral spray or nasal spray on their own or in combination with one other non-patch product.

Most people (95.4%) had discontinued treatment at the end of study period but data is not reported in the THIN data on whether smoking cessation had been successful or not. The proportion of discontinuation was similar for each initial NRT, suggesting there was no difference in this proportion between monotherapy and combination therapy.

## Discussion

There is currently little published information on the characteristics of people prescribed NRT or on NRT prescribing by GPs. This study provides information describing people prescribed NRT patch and combination regimens involving a patch by GPs in UK primary care.

The demographics of our study population were much as expected and consistent with the demographics of the overall UK smoking population [12] and the findings of Brose et al. [5]. The equal distribution of men and women in the study population is consistent with the overall prevalence of smoking in the UK, where the difference in smoking prevalence between men and women has decreased considerably over the years. In 2010, the prevalence of smoking was similar among men and women with 20% of men and 19% of women reporting smoking [12]. There were slight differences in the gender distribution across initial NRT, for example a higher proportion of males received initial NRT of patch and nasal spray (62.8%) or patch and gum (53.4%) compared to females.

The difference in the distribution of social deprivation for the study population compared with the overall THIN population might be reflective of the difference between the overall smoking population and the sub-group of people prescribed smoking cessation products. While smoking tends to be more prevalent among those in groups who work in routine and manual jobs [13], there may be a greater willingness for these people to seek out prescribed smoking cessation products if they qualify for free prescriptions. More affluent smokers may be more willing to access products over the counter, which have to be paid for out of pocket. The Townsend score distribution was similar across all initial NRT types.

NICE recommends three pharmacotherapies – NRT, bupropion and varenicline – as first-line therapy for smoking cessation [4]. The choice of pharmacotherapy within the NICE guidance is recommended to be a tailored choice arising from discussion between the individual seeking help and the advising health professional. There is currently no evidence to suggest that any specific form of single NRT is more effective than another form [14]. However, two recent network meta-analyses (NMAs) based on evidence from randomised controlled trials indicate that varenicline and combination NRT are more effective than single forms of NRT [15,16].

Our study is an incidence study – i.e. including people who had a first NRT patch prescription and then following them through the study period. However, in the entire NRT (patch + non-patch) population (N = 128,115) identified during the study period prior to study exclusion criteria being applied, only 14.7% (N = 18,838), had a history of bupropion or varenicline prescribed as a smoking cessation treatment, leading to their exclusion. This indicates that GPs appear to usually prescribe NRT as the initial smoking cessation treatment in primary care despite the NICE guidance on smoking cessation pharmacotherapy which recommends equal first-line positioning for NRT, varenicline and bupropion.

This study uses a first NRT patch prescription as the starting place – index medication – for our study. The majority of the study population (64.3%) received NRT patch monotherapy during their first recorded smoking cessation attempt. However, the proportion starting on combination therapy increased across the duration of the study period, from 25.7% of people identified in 2008 to 44.8% of people identified in 2011. This indicates that the use of combination NRT appears to be on the increase amongst GP prescribers.

The high rates of use of NRT patch monotherapy in second (50.2%) and third episodes (45.7%) is surprising as it appears that similar NRT strategies to the first episode are being employed in subsequent attempts, and it is unclear why the strategy of use of NRT patch monotherapy would be used again in this way.

The majority of the included study population had only one recorded smoking cessation attempt. However, 39.3% (N = 50,388) of people from the total patch and non-patch NRT cohort were excluded owing to a history of NRT products and 20.2% (N = 7,868) of the included study population started a subsequent NRT episode after their first episode. Although this is an incidence study, rather than a prevalence study, this rate of exclusion suggests that, if these two groups of people are considered together, a substantial overall proportion, 45.5% (58,256/128,115), of NRT study subjects do proceed to a second episode of NRT. Furthermore, this is likely to be an underestimate of the true rate of repeat NRT use since some of the first-line NRT patch users in the analysis may have already had NRT in over the counter settings, which would not be recorded in the THIN database.

The majority of people who received non-patch NRT as a first-line NRT received either an inhalator only (50.6%) or gum only (16.8%). These deliver ‘short term’ doses of nicotine, rather than the patch which delivers nicotine over a longer period. Furthermore, 13.6% received more than one type of non-patch NRT prescribed in combination. This is unexpected as there is no recommendation in the NICE guidelines for the use of exclusive non-patch NRT combinations [4].

We observed an average of one year between NRT episodes with a range of 91 to 1693 days. This is a longer interval than expected. NICE guidance for smoking cessation states that “*if a smoker's attempt to quit is unsuccessful using NRT, varenicline or bupropion, do not offer a repeat prescription within 6 months unless special circumstances have hampered the person's initial attempt to stop smoking, when it may be reasonable to try again sooner*” [4].

### Strengths and limitations of the research methods

The primary weakness of this study is that it was not possible to include treatment bought by people over the counter or NRT vouchers provided by smoking cessation clinics. The study may therefore overestimate the number of first-time NRT patch users identified in the analysis and underestimate the proportion of repeat NRT use in the study population. In addition, as THIN data is based on records of prescribing, therapy prescribed does not necessarily equate to therapy dispensed or take account of patient compliance with treatment.

Reasons for changes in treatment are not captured in THIN. The success of a patient’s smoking cessation attempt is unavailable. It is also worth noting that records of the NRT oral spray had a shorter amount of follow-up time available as oral sprays were not authorised until November 2010 [17].

## Conclusions

This real world observational study provides, for the first time, descriptive information about people prescribed NRT by GPs to support smoking cessation. It provides valuable data for the evaluation of GP NRT prescription patterns in UK primary care as well as supplying a detailed resource based on real world evidence to inform economic modelling. It also provides useful information on the impact of NICE guidelines [4] on prescribing during the study period.

The majority of the first recorded smoking cessation attempts with NRT patch between 2008 and 2011 were monotherapy. However, the rate of initiation with combination therapy has increased over time to 45% of all episodes involving initiation with an NRT patch. Furthermore, the study indicates that in a large cohort of over 128,000 NRT users (patch and non-patch), bupropion and varenicline are rarely prescribed prior to NRT use. Given evidence from recent NMAs based on RCTs [15,16] that varenicline is more effective than NRT monotherapy and is as effective or more effective than combination NRT involving a patch [15,16], the low rate of GP varenicline prescribing, as suggested in the present study, requires further evaluation from both a policy and research perspective.

While further analysis should explore the factors associated with initial prescribing decisions and changes in treatment, given the predominance of NRT patch monotherapy, there is also a need for health policy makers and service commissioners to ensure that GPs provide equality of patient access to and uptake of all NICE-recommended smoking cessation pharmacotherapies.

## Competing interest

The study was funded by Pfizer Ltd. Ian Lockhart was an employee of Pfizer Ltd. at the time the study was completed. Michelle Johnson was an employee of Cegedim Strategic Data Medical Research Ltd at the time the study was completed and Pippa Anderson is an employee of the Swansea Centre for Health Economics, Swansea University.

## Authors’ contributions

IL conceived of the study, and participated in its design and coordination. IL was an employee of Pfizer Ltd at the time the study and manuscript were completed.PA and MJ participated in the design and execution of the study. MJ performed the statistical analysis. PA drafted the manuscript. All authors read and approved the final manuscript.

## Pre-publication history

The pre-publication history for this paper can be accessed here:

http://www.biomedcentral.com/1471-2296/15/47/prepub
